# Worldly Grounding and the Puzzles

**DOI:** 10.1007/s11098-025-02378-w

**Published:** 2025-09-06

**Authors:** Fabrice Correia

**Affiliations:** Department of Philosophy, University of Geneva, Geneva, Switzerland

**Keywords:** Grounding, Puzzles of grounding

## Abstract

Starting with Kit Fine’s article “Some Puzzles of Ground” (2010), grounding has been argued to give rise to serious puzzles. The way the puzzles should be handled, I contend, depends on whether the concepts of grounding involved in the puzzles are worldly or representational. Here I focus on worldly grounding, and I argue that the formally best understood worldly concepts of worldly grounding, which I dub “weak-first”, escape all the currently known puzzles, including new puzzles that I introduce in due course.

Starting with Kit Fine’s article “Some Puzzles of Ground” (2010a), grounding has been argued to give rise to serious puzzles: plausible ground-theoretic principles, typically together with auxiliary premises which look harmless, have been shown to generate inconsistencies. It is my view that the appropriate attitude towards these puzzles requires one to take seriously the distinction between *worldly* and *representational* grounding. The way the puzzles should be handled, I contend, depends on whether the concepts involved are of one sort or of the other. In this paper, I wish to focus on worldly concepts. I will more specifically focus on concepts of grounding that are both worldly and “weak-first”. One might wonder whether this latter qualification is a substantial restriction. I do not have a firm opinion on this question, but I do know that the restriction is immaterial “in practice”: *all* the logics of worldly grounding that have been devised so far target concepts of grounding that *are* weak-first.

Here is the plan. I first present what I call the *Finean puzzles* (§1), which include, but are not limited to, the puzzles discussed by Fine (2010a). I then identify a general strategy for escaping these puzzles which I take to be the right one on the assumption that the concepts of grounding at stake are worldly (§2). The strategy is later implemented for concepts of grounding that are both worldly and weak-first (§6). In the intermediate sections, I characterize the worldly vs. representational distinction (§3), I define weak-first grounding (§4), and I identify a set of principles for weak-first worldly grounding that plays a crucial role in the resolution of the Finean puzzles (§5). The simplest, and perhaps also the most impressive one, among the Finean puzzles is a puzzle introduced by Krämer (2013) which involves higher-order quantification. Before moving on to the last two sections, where I discuss non-Finean puzzles, I criticize a way of escaping Krämer’s puzzle that has been recently suggested (§7). Finally, in §8 I discuss puzzles involving truth-teller and liar sentences, and in §9 a puzzle for concepts of *immediate* grounding. Whereas the resolution of the former puzzles, just like that of the Finean puzzles, crucially invokes the property of being both worldly and weak-first, the resolution of the latter only invokes the property of being worldly.

## The Finean puzzles

The Finean puzzles all have the following general shape: the asymmetry or irreflexivity of partial grounding (≺) or full grounding (<) is shown to be inconsistent with plausible ground-theoretic principles (and in some cases auxiliary premises).^1^ Following orthodoxy, I take ≺ and < to be sentential operators, the former binary and the latter “(one or many)-to-one”: the basic claims involving it take the form $\Delta < \phi$ where ϕ is a sentence and Δ is a nonempty list of sentences. Even though the puzzles are often formulated on the assumption that the concepts of grounding involved are factive (the grounds and the groundees are required to hold for the grounding connection to obtain), for simplicity I assume that < and ≺ are non-factive.^2^ Nothing of substance should follow from this decision.

The relevant notions of asymmetry and irreflexivity can be spelled out as follows, where *p*, *q*, *P* and *Q* are sentential variables, the former two singular and the latter two plural (and where each defining condition is understood as having its variables bound by outer universal quantifiers; I will often omit outer universal quantifiers for the sake of readability):

$${\textbf {Asymmetry}}_\prec $$**:** $$\lnot (p \prec q \wedge q \prec p)$$
$${\textbf {Irreflexivity}}_\prec $$**:** $$\lnot (p \prec p)$$
$${\textbf {Asymmetry}}_<$$**:** $$\lnot (P, p< q \wedge Q, q < p)$$
$${\textbf {Irreflexivity}}_<$$**:** ¬(P,p<p)

Of course, $\hbox {Asymmetry}_\prec$ entails $\hbox {Irreflexivity}_\prec$, and $\hbox {Asymmetry}_<$ $\hbox {Irreflexivity}_<$. Given the orthodox assumption that ≺ is transitive, $\hbox {Asymmetry}_\prec$ is equivalent to $\hbox {Irreflexivity}_\prec$. And likewise, $\hbox {Asymmetry}_<$ is equivalent to $\hbox {Irreflexivity}_<$ given the orthodox assumption that < is transitive in the sense that the following always holds:

$$(P< p \wedge p, Q< q) \rightarrow P, Q < q$$

If ≺ is defined in terms of < in the natural way (a partial ground $\hbox {is}_{\text {df}}$ either a full ground or a proper part of a full ground), then $\hbox {Asymmetry}_\prec$ is equivalent to $\hbox {Asymmetry}_<$ and $\hbox {Irreflexivity}_\prec$ to Irreflexivity$_<$. Thus, taking ≺ to be defined in terms of < in the suggested way and taking < to be transitive in the suggested sense, the four conditions listed above are all equivalent.

Let me run through a representative set of four puzzles. They are all formulated in terms of partial grounding, but they could easily be formulated in terms of full grounding, possibly in some cases with slight modifications, without loosing their force.

The first puzzle assumes the asymmetry of ≺ and establishes a contradiction from the following three assumptions, where *T* is a predicate for propositional truth and $\langle \phi \rangle$ is used for “the proposition that ϕ”:

$${\textbf {Existence}}_1$$**:** $$\exists y (y = \langle \exists x Tx\rangle )$$
*T***-Grounding:** $$(a = \langle \phi \rangle ) \supset (\phi \prec Ta)$$
∃x**-Grounding:** $$\phi [a/x] \prec \exists x \phi $$

$\hbox {Existence}_1$ says that there is such a thing as the proposition that there is a true proposition; *T*-Grounding says essentially that the truth of the proposition that ϕ is partly grounded in its being the case that ϕ; and ∃x-Grounding says that existential generalizations are partly grounded in their instances. The derivation of the contradiction can be represented by means of the following semi-formal natural deduction proof (from Fine (2010a), adapted by Krämer (2020)):

**Figure:**
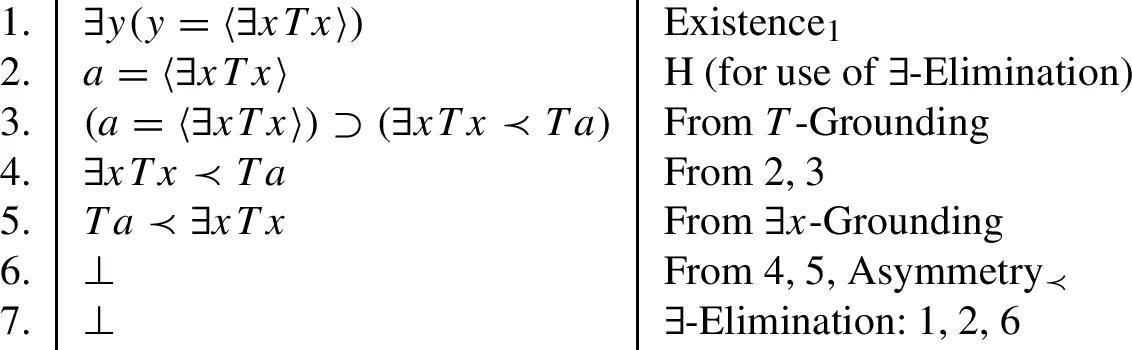

The second example involves disjunction instead of existential quantification. It still invokes *T*-Grounding, but appeals to the following two principles in place of $\hbox {Existence}_1$ and ∃x-Grounding:

$${\textbf {Existence}}_2$$**:** $$\exists y (y = \langle 0 = 0 \vee Ty\rangle )$$
∨**-Grounding:** $$\phi \prec \psi \vee \phi $$

Here is the derivation (from Krämer (2020), inspired by Fine (2010a)):

**Figure:**
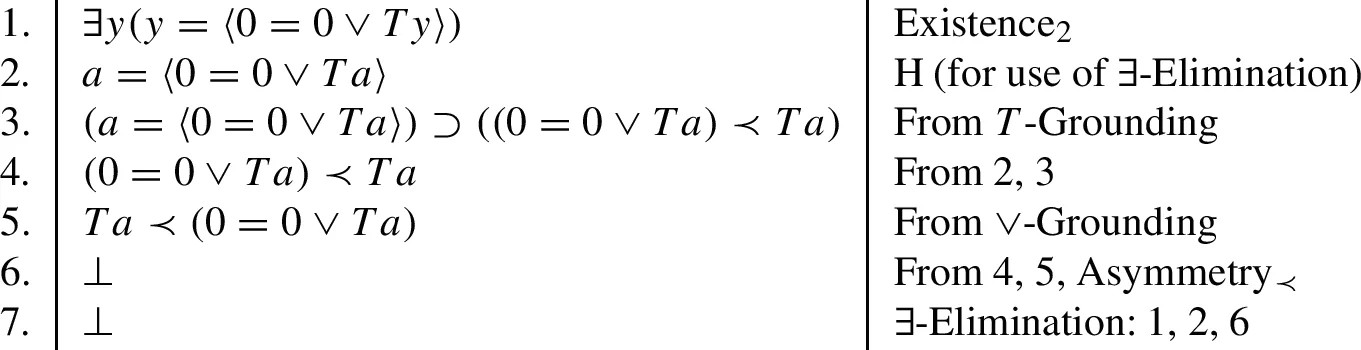

These two examples assume that the range of the first-order quantifiers comprises *propositions*. The following two examples are free from such an assumption. They both involve higher-order rather than first-order quantification, and they invoke the following principle instead of ∃x-Grounding:

∃p**-Grounding:** $$\phi [\psi /p] \prec \exists p \phi $$

One of the examples invokes a higher-order version of *T*-Grounding, formulated using the truth operator $\textsf {T}$ instead of the truth predicate *T*:

$$\textsf {T}$$**-Grounding:** $$\phi \prec \textsf {T}\phi $$

The derivation goes as follows (from Correia (2011)):

**Figure:**
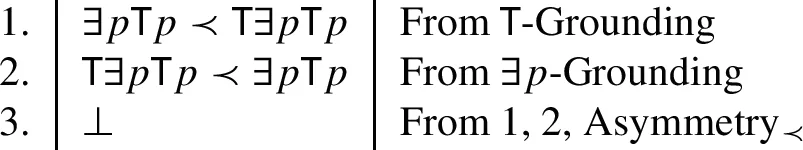

The fourth example is even simpler. The derivation only makes use of ∃p-Grounding, and establish that this principle is inconsistent with Irreflexivity$_\prec$ (from Krämer 2013):

**Figure:**
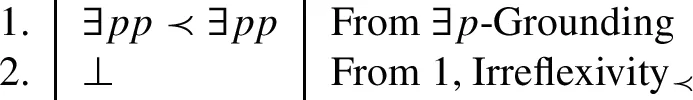

(We get 1 from ∃p-Grounding by taking *p* for ϕ and ∃pp for ψ.)

## A strategy

Each of the four puzzles involves a structural feature that orthodoxy attributes to ≺: irreflexivity in the case of the fourth derivation, and the stronger feature of asymmetry in the case of the other derivations. In light of the fourth puzzle, it is clear that if one wishes to stick to the view that ≺ is irreflexive, one has to abandon ∃p-Grounding. If this latter principle is given up, not only the fourth derivation but also the third one will be undermined. It is natural to think that if ∃p-Grounding is to be given up, then the same is true of ∃x-Grounding and ∨-Grounding. If these principles are in turn abandoned, then the first two derivations will also be undermined. Thus, a strategy suggests itself in order to save orthodoxy about the structural features of ≺: get rid of the ground-theoretic introduction principles at work in the derivations.

The strategy can actually be generalized to *all* the Finean puzzles that have been discussed in the literature (and obvious variants thereof). All these puzzles, be they about ≺ or <, involve ground-theoretic introduction principles. As we saw, some puzzles involve an introduction principle for existential quantification, and some involve an introduction principle for disjunction. The other Finean puzzles that do not involve such introduction principles involve an introduction principle for universal quantification or an introduction principle for conjunction. The generalized strategy is straightforward: reject all these introduction principles. This is the strategy I wish to implement in this paper.

I hasten to stress that I do not think that this is the only strategy which can be implemented. I am a pluralist about grounding, I take it that there are several concepts of grounding and that some of them significantly differ from others in their logical features. In Correia (2014), I argue that there is a concept of grounding, which I dub *logical grounding*, which conforms to the unrestricted introduction principles and which is *not* irreflexive. (Slightly anticipating the introduction of the distinction, it is a concept I take to be *representational* as opposed to *worldly*.) What I wish to do in what follows is argue that there is another concept of grounding—or, more cautiously, a family of such concepts—for which the above-mentioned strategy is appropriate.

The concepts of grounding in question are those that are both *worldly* and *weak-first*. I introduce the distinction between worldly and representational grounding in §3 and weak-first grounding in §4.

## Worldly vs. representational grounding

The distinction between worldly and representational (aka conceptual) grounding was first introduced in Correia (2010).^3^ The distinction is characterized in terms of the concept of *factual equivalence*, i.e., the concept expressed by the sentential operator

for it to be the case that … is for it to be the case that …

which I shall abbreviate to

$$\ldots \equiv \ldots $$

If ϕ and ψ are truth-evaluable sentences, $\phi \equiv \psi$ says that ϕ and ψ describe the world as being the same way. A concept of full grounding is said to be *worldly* iff it is “closed” or “invariant” under factual equivalence. More explicitly, using again < for full grounding, the concept expressed by < is said to be worldly iff the following two conditions always hold:

If P<p and p≡q, then P<q

If $p^{\prime },p^{\prime \prime },...<p$ and $p^{\prime }\equiv q^{\prime }$ and $p^{\prime \prime }\equiv q^{\prime \prime }$ and ..., then $q^{\prime },q^{\prime \prime },...<p$

A concept of full grounding is said to be *representational* iff it is not worldly. These definitions can be adapted to partial grounding in the obvious way. Note that if a concept of full grounding is worldly, then its natural partial counterpart must also be worldly.^4^

Worldliness has a direct impact on the logic of grounding, in particular on the introduction principles for disjunction and conjunction. Consider for instance the version of these principles for full grounding (the argument carries over to partial grounding). In unrestricted form, these are the following principles:^5^

p<p∨q       q<p∨q       p,q<p∧q

Plausibly, $(p \vee p) \equiv p$ always holds. Granted that this is the case and that < is worldly, from each disjunction introduction principle one can infer that < is reflexive in the standard sense—i.e., that p<p always holds. The same conclusion can be derived from the plausible view that $(p \wedge p) \equiv p$ always holds, the assumption that < is worldly and the conjunction introduction principle. Now full grounding, whether worldly or not, is certainly *not* reflexive. Hence, granted that both $(p \vee p) \equiv p$ and $(p \wedge p) \equiv p$ always hold, the unrestricted introduction principles for worldly grounding must be rejected.

This argument against the unrestricted principles for worldly grounding is explicitly premised on the view that $(p \vee p) \equiv p$ and $(p \wedge p) \equiv p$ always hold. As I have indicated, I take this view to be plausible. In Correia (2016), I advocate a whole *logic* of factual equivalence that validates this view. Space is lacking for a useful summary, so I will here simply assume that the view is correct.^6^

The previous considerations have, of course, an immediate bearing on the Finean puzzles. Some of the derivations giving rise to the puzzles invoke unrestricted disjunction introduction for grounding (full or partial), other derivations invoke unrestricted conjunction introduction. By the previous considerations, these derivations cannot be used to generate puzzles for *worldly* grounding.

What about the remaining Finean puzzles? As we saw, they involve unrestricted introduction principles for existential and universal quantification. Given the logical similarity between existential quantification and disjunction on one hand, and universal quantification and conjunction on the other hand, it is natural to doubt, or at least to be wary of the view, that the principles in question hold if interpreted as concerning worldly grounding. Given that these principles are questionable, the derivations giving rise to these puzzles are also harmless if taken to concern worldly grounding.

We are on a good track. But we are not done yet. Granted that the relevant introduction principles for worldly grounding fail, or at least are questionable, in unrestricted form, surely some *restricted* versions of these principles *do* hold. Now for all we know at this stage, the derivations that give rise to the Finean puzzles might well be valid if construed as invoking acceptable restricted principles instead of the unrestricted principles. We need a principled account of the introduction principles for worldly grounding that can be shown *not* to licence these derivations. Such an account will be given in §5, and an argument to the effect that the principles do not licence the derivations in §6.

## Weak-first grounding

A concept of full grounding is *weak-first* iff the following two conditions are satisfied:

1. It is connected to a concept of *weak* full grounding in a specific way, to be spelled out below;
2. This weak concept is reflexive and obeys unrestricted introduction principles for ∨, ∧, ∃ and ∀ to be specified below.

Associated with each full weak-first concept—which we dub “strict” to distinguish it from the weak concept it is associated with—there is a partial concept, the one that is defined in the natural way in terms of the full concept, which also counts as a weak-first concept. Every weak-first concept is either a strict full concept or a partial concept associated to a strict full concept in the way just mentioned.

Let me unpack conditions (1) and (2).

Let < be a concept of strict full grounding that is weak-first, and let ⩽ be the weak concept associated with it as per condition (1). To formulate the connection between < and ⩽ mentioned in this condition, it is useful to invoke the natural partial counterpart ≼ of ⩽ (p≼q $\hbox {iff}_{\text {df}}$ for some *P* such that p∈P, P⩽q):

C $$\begin{aligned} P < p\text { iff }P \leqslant p\text { and for no }q\in P\text { is it the case that }p \preccurlyeq q \end{aligned}$$

As Fine (2012: 52) puts it: a strict ground is a weak ground which cannot be “reversed”.

The unrestricted introduction principles for ∨ and ∧ mentioned in condition (2) are as follows:

$$p \leqslant p \vee q$$       $$q \leqslant p \vee q$$       $$p, q \leqslant p \wedge q$$

Those for ∃ and ∀ respect the logical similarity between ∃ and ∨ and that between ∀ and ∧ and are inspired by the principles just formulated. The details are a matter of discussion.

Here are two natural candidates for the *first-order* quantifiers, as close it is possible to get to the ∨- and the ∧-introduction principles:

$$\phi [a/x] \leqslant \exists x \phi $$

$$\phi [a'/x], \phi [a''/x], \ldots \leqslant \forall x \phi $$

where $a^{\prime }$, $a^{\prime \prime }$, … name all the objects that there are

Following Fine (2012), it may be argued that the ground in the ∃-introduction principle should be modified by adding the condition “*a* exists”.^7^ Also following Fine (2012), one may wish to “internalize” the condition on names in the ∀-introduction principle, so that the ground includes the condition “$a^{\prime }$, $a^{\prime \prime }$, ... are all the objects that there are”. And still further variants can be advocated. Which versions of the principles are taken on board will be immaterial for our purposes, and so, for the sake of simplicity, I will ignore the versions distinct from the simple versions listed above.

When it comes to higher-order quantifiers, we enter into widely unexplored territory, but the general idea is to treat them like the first-order quantifiers. To illustrate with the sentential quantifiers—which are at work in two of the puzzles presented in §1—I will take the introduction principles to be:

$$\phi [\psi /p] \leqslant \exists p \phi $$

$$\phi [\psi '/p], \phi [\psi ''/p], \ldots \leqslant \forall p \phi $$

where $\phi [\psi '/p], \phi [\psi ''/p], \ldots$ are all the relevant instances

What the “relevant instances” are is a matter of discussion, but for our purposes it will not be necessary to be more specific.

Two remarks before moving on to the next section.

Nothing in the characterization of weak-first concepts of grounding demands that these concepts should be worldly. Some studies in the literature target weak-first concepts that *are* worldly (Correia (2010, 2023), Fine (2012) (semantics), Correia and Skiles (2019)); other studies target weak-first concepts that *are not* (Fine (2012) (proof theory) and its sequel deRosset and Fine (2023)); one study, developing views advocated in Correia (2010) and Fine (2012), is explicitly neutral on whether the weak-first concept it targets is worldly or representational (Lovett, 2020a).^8^

(C) is suggestive of a *definition* of < in terms of ⩽, and it is because of this that I chose the label “weak-first” for the concepts characterized above. But one should not take the suggestion too seriously: (C) is intended to be just a biconditional. In Correia and Skiles (2019), Skiles and I *do* define (“give an account of”) strict full grounding in terms of weak full grounding along the lines of (C). This is also the view Fine (Fine (2012): 52-53) is inclined to adopt, and a view that I do adopt in Correia (2023). By contrast, I do not take this approach in Correia (2010), and neither does Lovett (2020a, 2020b).

## Introduction principles for weak-first worldly grounding

Let < be a concept of strict full grounding that is weak-first, let ⩽ be the associated weak full concept, and let ≺ and ≼ be their respective partial counterparts. Let me use  for the negation of ...≼... . Thanks to (C), from the unrestricted ∨- and ∧-introduction principles for ⩽ one can immediately derive the following restricted principles for < and ≺:

**Figure:**
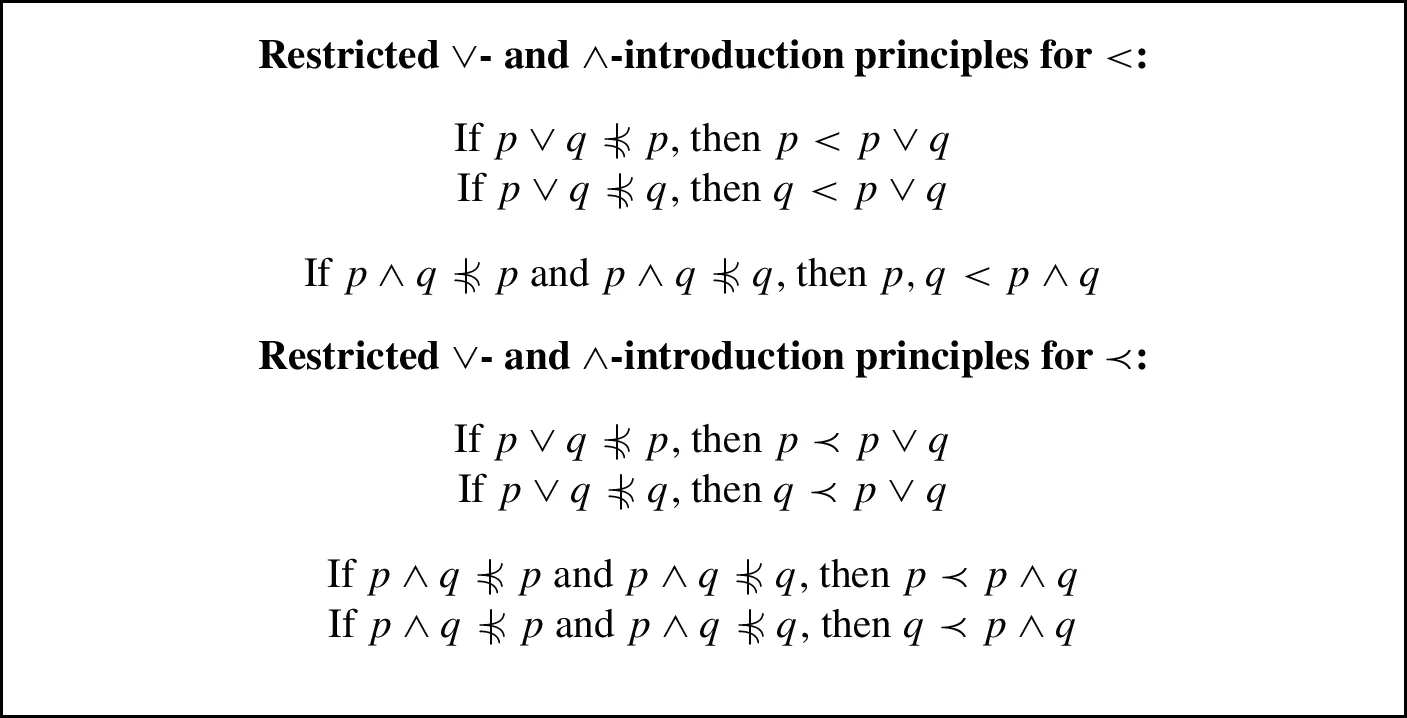

And from the unrestricted first-order ∃- and ∀-introduction principles for ⩽, one can derive the following restricted principles for < and ≺:

**Figure:**
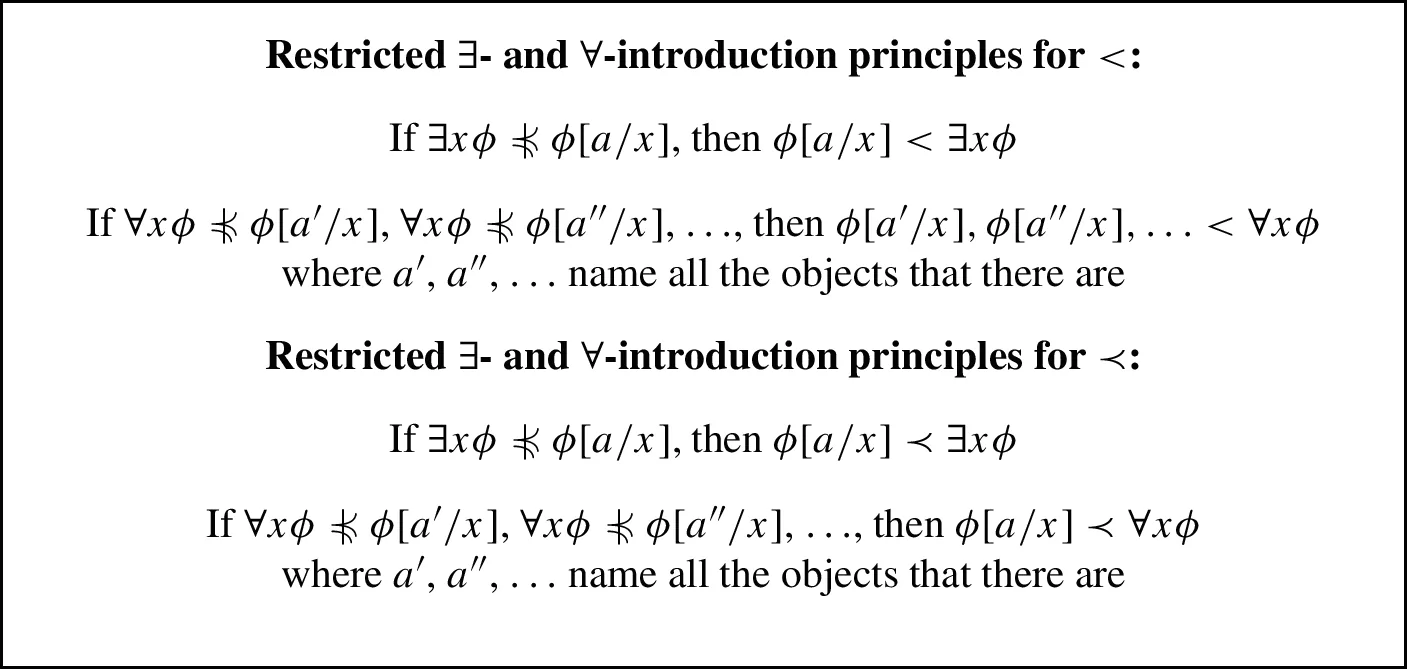

And likewise for the higher-order quantifiers.

Importantly, so far nothing rules out that < and ≺ satisfy, in addition to these restricted principles, the original unrestricted principles. Whether < is worldly or representational makes a crucial difference in this connection.

Suppose first that < is worldly. Then ≺ is also worldly. Reasoning as in §3, we get to the conclusion that the relevant unrestricted ∨- and ∧-introduction principles for < and ≺ are false, and that the relevant unrestricted ∃- and ∀-introduction principles for < and ≺ are questionable. Hence, in this case, the rational attitude is to give assent only to the restricted principles for < and ≺.

Suppose now that < is representational. In this case, of course, the argument from §3 against the unrestricted ∨- and ∧-introduction principles is ineffective. It actually seems that no principled reason rules out that < satisfies the unrestricted principles in which it features. If it does satisfy them, then ≺ will satisfy the corresponding unrestricted principles. The concept of strict full grounding studied in Fine (2012) (proof theory) and deRosset and Fine (2023) is weak-first, representational, and *does* satisfy the unrestricted introduction principles in question.

In what follows, I will leave representational grounding aside and focus exclusively on weak-first worldly grounding. The aim will be to show that weak-first worldly grounding, with its restricted introduction principles, escapes the Finean puzzles, as well as other puzzles that I will introduce in due course.

## Back to the Finean puzzles

The previous material should make clear, at least to some extent, how the Finean puzzles are to be handled if we take them to concern weak-first worldly grounding.^9^ In this section, let me use < for a strict full concept of grounding that is both weak-first and worldly, ⩽ for the associated weak full concept, and ≺ and ≼ for their respective partial counterparts.

Importantly, all the asymmetry and irreflexivity principles listed in §1 are provably satisfied. $\hbox {Asymmetry}_<$ is satisfied because < is weak-first: assuming for reductio P,p<q and Q,q<p, by condition (C) we get  and Q,q⩽p, and by the latter and the definition of ≼ we get q≼p—contradiction. $\hbox {Irreflexivity}_<$ follows from $\hbox {Asymmetry}_<$ by logic. Asymmetry$_\prec$ and Irreflexivity$_\prec$ then follow by the definition of ≺ in terms of <. Thus, questioning the use of these principles in order to escape the Finean puzzles is not even an option.

Let me first focus on the simplest Finean puzzle, the one put forward in Krämer (2013). I reproduce the corresponding derivation here for convenience:

**Figure:**
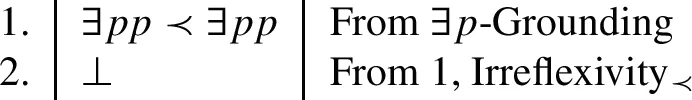

For the record, ∃p-Grounding is the unrestricted higher-order ∃-introduction principle for ≺:

$$\phi [\psi /p] \prec \exists p \phi $$

As argued in §5, since ≺ is both weak-first and worldly, one cannot rely on this principle. Accordingly the derivation is blocked.

What we have in place of ∃p-Grounding is the following restricted principle:

If , then $$\phi [\psi /p] \prec \exists p \phi $$

Can we use it to secure the first line of Krämer’s derivation? The answer is negative. Taking *p* for ϕ and ∃pp for ψ as in the original argument, what we get from the restricted principle is the following conditional:

If , then $$\exists p p \prec \exists p p$$

In order to get from here to the first line of the derivation, we would need to establish that the antecedent of the conditional is true. But since ⩽ is reflexive, the definition of ≼ in terms of ⩽ guarantees that ≼ is also reflexive and hence that the antecedent is *false*. Thus, the puzzle cannot be reinstated using the restricted principle instead of the unrestricted principle.

Let me move on to the derivation taken from Correia (2011):

**Figure:**
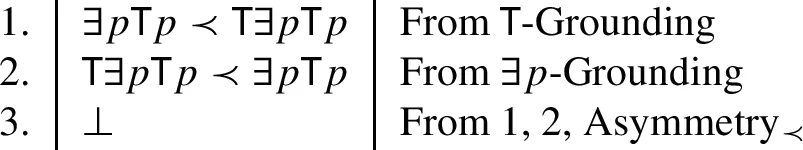

This derivation, like the previous one, invokes ∃p-Grounding, and so the verdict is the same: since ≺ is both weak-first and worldly, appeal to this principle is illegitimate, and therefore the derivation is defective.

Can we use the restricted counterpart of ∃p-Grounding to secure the relevant line of the derivation? The answer is, as with the previous derivation, negative, but the reason is significantly different. The relevant instance of the restricted principle is the following conditional:

If , then $$\textsf {T}\exists p \textsf {T}p \prec \exists p \textsf {T}p$$

In order to get from here to the second line of the derivation, we would need to establish that the antecedent of the conditional is true. This time, it is not a structural property of ⩽ that implies, together with relevant definitions, the falsity of the antecedent, but the first premise of the derivation and the fact that < is stronger than ⩽—which follows from condition (C). To see this, assume the first premise of the derivation, i.e., that $\exists p \textsf {T}p \prec \textsf {T}\exists p \textsf {T}p$. By the definition of ≺ in terms of < we then have that $P, \exists p \textsf {T}p < \textsf {T}\exists p \textsf {T}p$ for some *P*. Since < is stronger than ⩽, it follows that $P, \exists p \textsf {T}p \leqslant \textsf {T}\exists p \textsf {T}p$ for some *P*. By the definition of ≼ in terms of ⩽, we can infer that $\exists p \textsf {T}p \preccurlyeq \textsf {T}\exists p \textsf {T}p$.

A quick inspection of the other derivations discussed in §1 shows that with each of them the situation is exactly as with the derivation just discussed: a premise is said to be justified by an unrestricted introduction principle, whereas appeal to this principle is illegitimate; and appealing to the corresponding restricted principle is ineffective to justify the premise in question, because its relevant instance, which is a conditional having the premise as consequent, has an antecedent that can be shown to be false given some other premise(s) of the derivation.

All the other Finean derivations that can be found in the literature give rise to the same sort of situation. Hence, the Finean puzzles do not arise for weak-first worldly concepts of grounding.

Lovett (2020b), drawing on his previous study of the logic of grounding (2020a), advocates a way of handling the Finean puzzles that is superficially similar to the one I have just developed, but which I take to be defective. Lovett’s proposal has two parts. Let me first present and criticize them in turn, and then summarize the main differences between our respective approaches.

In the first part of his proposal, Lovett introduces a set of unrestricted introduction principles for ⩽ that concern notions at work in the versions of the Finean puzzles he discusses—including disjunction, conjunction, existential quantification, universal quantification and truth (2020b: 2556).^10^ He then suggests to define < in terms of ⩽ along the lines of condition (C) (2557) and notes that (i) all that can be derived from the unrestricted introduction principles for ⩽ plus condition (C) is a collection of restricted introduction principles for strict grounding and (ii) with these restricted principles alone one cannot derive the puzzles (2557-2558). So far so good. Like me, he puts forward a weak-first conception of strict grounding and, like me, he notes that the view that grounding is weak-first, by itself, does not allow one to derive the problematic introduction principles. Yet he goes one step further and takes it that, at this stage, he has a solution to the puzzles (*ibid*).

But he does not. Strict grounding may be weak-first *and yet* conform to the problematic, unrestricted principles. As I stressed at the end of §5, there *are* accounts of strict grounding according to which the concept has both features (namely, the accounts in Fine (2012) (proof theory) and deRosset and Fine (2023)).

The second part of Lovett’s proposal is meant to strengthen the first part. We already have a solution to the puzzles, Lovett thinks (wrongly, as I have just argued). But it is “mainly technical”, no “deep motivation, independent of the paradoxes” for restricting the introduction principles for strict grounding was given apart from the idea that strict grounding should be defined in terms of weak grounding along the lines of (C) with the background of the unrestricted principles for weak grounding (2560). But it would be “nice”, he adds, to have a further motivation for restricting the principles (*ibid*). The motivation he puts forward is this: take grounding to be worldly.

My objection against this part of Lovett’s proposal is simply that taking strict grounding to be worldly cannot provide a *further* motivation for ruling out the unrestricted introduction principles, since, as I argued while discussing the first part of Lovett’s proposal, taking strict grounding to be weak-first does not provide a motivation for doing it in the first place!

Like Lovett, I invoke the properties of being weak-first and of being worldly, but I do it in a very different way. On my view, appealing to the worldly character of strict grounding provides *the* argument—as opposed to an additional argument—for the conclusion that the concept fails to conform to the unrestricted principles (see §3). And I see the appeal to the weak-first character of strict grounding, not as providing an argument for that conclusion, but as providing, *given the background view that strict grounding is worldly and hence fails to conform to the unrestricted principles*, (a) a principled account of the restrictions to which the principles are subject (see §5) and, on top of this, (b) a principled account of the way in which these restrictions block the puzzles (see the beginning of §6).

## Fritz’s Fregean move

In response to Krämer (2013) puzzle, Fritz (2020) explores the idea of replacing ∃p-Grounding by a “Frege-style” principle that expresses the same core idea—a principle that I will call *E-Grounding*. This principle, unlike ∃p-Grounding, does not immediately give rise to a violation Irreflexivity$_\prec$. Fritz argues that it does give rise to a violation of $\hbox {Irreflexivity}_\prec$ or $\hbox {Asymmetry}_\prec$ under substantial assumptions about lambda abstraction, but he then sketches a general theory on which these assumptions fail. Could Fritz’s Fregean move provide a way of escaping higher-order Finean puzzles in general? This is what the conclusion of Fritz’s paper suggests. But I argue that it does not: I argue that *E*-Grounding generates violations of $\hbox {Irreflexivity}_\prec$ and $\hbox {Asymmetry}_\prec$ irrespective of any view about lambda abstraction. And I also argue that weak-first worldly grounding escapes the puzzles generated by *E*-Grounding in the very same way it escapes Krämer’s puzzle.

The Fregean idea invoked by Fritz is that the quantifiers are to be understood as “higher-order” properties: “there are *F*s”, according to this idea, expresses that the property of being *F* has the property of being exemplified. The principle that Fritz suggests as a replacement of ∃p-Grounding, *E*-Grounding, is formulated as follows:^11^

*E***-Grounding:** $$\forall X \forall p (Xp \prec EX)$$

In *E*-Grounding, *X* is a variable of the grammatical type of a monadic sentential operator, *p* a sentential variable and *EX* is the Fregean way of saying that for some *p*, *Xp*.^12^

In Krämer’s argument, the instantiation of ∃p-Grounding yields $\exists p p \prec \exists p p$. Fritz translates ∃pp into the Fregean Eλqq, and for the purpose of mimicking Krämer’s argument, starting from *E*-Grounding, he accordingly instantiates *X* with λqq and *p* with Eλqq to obtain the following sentence:

$$\lambda qq E\lambda qq \prec E\lambda qq$$

This is not a counterexample to $\hbox {Irreflexivity}_\prec$. We would get from this a counterexample to $\hbox {Irreflexivity}_\prec$ if we assumed that $\lambda qq E\lambda qq = E\lambda qq$ (where = is a higher-order version of standard first-order identity) and the relevant instance of Leibniz’s Law. And we would get a counterexample to $\hbox {Asymmetry}_\prec$ if, following Fine (2012), we assumed that $E\lambda qq \prec \lambda qq E\lambda qq$. What Fritz does in the rest of the paper is introduce and briefly discuss a general view on lambda abstraction that falsifies both principles.

Fritz’s suggested way of instantiating *E*-Grounding does not conflict with $\hbox {Irreflexivity}_\prec$ or $\hbox {Asymmetry}_\prec$ unless substantial principles are taken to hold. But there are other ways of instantiating *E*-Grounding which lead to trouble—in fact, as much as do the corresponding ways of instantiating ∃p-Grounding. Let me run through a few illustrations.

Let *O* be an arbitrary monadic sentential operator. One gets the following sentence by instantiating *E*-Grounding:

**Figure:**
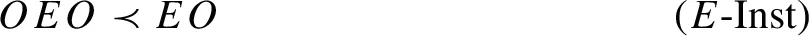

Correspondingly, starting from ∃p-Grounding one get the following sentence:

**Figure:**
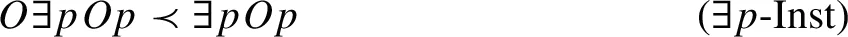

Suppose we take *O* to be *transparent*, where this means that ∀p(Op=p) holds. Perhaps Fritz’s operator λqq is transparent, perhaps it is not. Be that as it may, other operators may plausibly be taken to be transparent. The operator “it is the case that” is an example that comes to mind. We could perhaps even introduce an operator by *stipulating* that it is transparent. Given that *O* is transparent, we have OEO=EO, and by *E*-Inst and the relevant instance of Leibniz’s Law we get a violation of $\hbox {Irreflexivity}_\prec$. Likewise, given that *O* is transparent, we have $O\exists p Op = \exists p Op$, and by ∃p-Inst and the relevant instance of Leibniz’s Law we get again a violation of $\hbox {Irreflexivity}_\prec$.

Other operators yield violations of $\hbox {Irreflexivity}_\prec$ or $\hbox {Asymmetry}_\prec$ together with substantial ground-theoretic principles, but in a way that concerns *all* the logics of grounding I know of that deal with the interaction between grounding and the truth-functional connectives—all the *impure* logics of grounding, to use terminology that has become common.

Let me elaborate on just one example.^13^ Let the monadic sentential operator ∞ be defined as follows: $\infty \phi := \lnot \lnot \phi$. (Alternatively, we could stipulate that $\forall p(\infty p = \lnot \lnot p)$.) All the impure logics of *representational* grounding I know of validate the principle $p\prec \lnot \lnot p$,^14^ and hence the following principle:

$$p\prec \infty p$$

From this we get:

$$E\infty \prec \infty E\infty $$

From *E*-Inst we get:

$$\infty E\infty \prec E\infty $$

This is a violation of $\hbox {Asymmetry}_\prec$. We also get a violation of $\hbox {Asymmetry}_\prec$ if we invoke ∃p-Inst instead of *E*-Inst and $\exists p \infty p$ instead of E∞.

Let us now assume that ≺ is *worldly*. It is hard to resist the view that $p\equiv \lnot \lnot p$ always holds.^15^ From this we get that $p\equiv \infty p$ always holds. And from this we get:

$$E\infty \equiv \infty E\infty $$

Given the previous instance of *E*-Inst and the fact that ≺ is worldly, we get a violation of $\hbox {Irreflexivity}_\prec$. We also get a violation of $\hbox {Irreflexivity}_\prec$ if we invoke ∃p-Inst instead of *E*-Inst and $\exists p \infty p$ instead of E∞.

Thus, Fritz’s Fregean move is not an option to escape higher-order Finean puzzles: *E*-Grounding gives rise to such puzzles as well as ∃p-Grounding.

The previous material should make clear, at least in great part, how the puzzles are to be handled if taken to concern weak-first worldly grounding. If < is weak-first, then its weak counterpart ⩽ will behave re Fregean existential quantification as it does re standard existential quantification and so the following counterpart of *E*-Grounding will hold:

$$\forall X \forall p (Xp \leqslant EX)$$

The following restricted introduction principle for < will follow:



Granted that < is not only weak-first but also worldly, we will not be entitled to take the corresponding unrestricted principle—namely, *E*-Grounding—to hold. But the restricted principle, if understood as concerning worldly grounding, cannot be used to generate the violations of Irreflexivity$_\prec$ discussed above.

To see this, consider the relevant kind of instantiation where *O* is an arbitrary monadic sentential operator:



Suppose first that *O* is transparent. Then EO=OEO and so we get:



But since ≼ is reflexive, the antecedent of the conditional is false, and therefore we cannot detach its consequent. Suppose now that *O* is ∞, so that we have:



Assuming as before $E\infty \equiv \infty E\infty$, from this and the fact that ≺ is worldly we can infer the following conditional:



In order to detach the consequent we would need to establish . But there is room for denying it. Consider the following double negation introduction principle for ⩽:

1 $$\begin{aligned} p\leqslant \lnot \lnot p \end{aligned}$$

All the impure logics of weak-first grounding I know of, whether they target worldly concepts or representational concepts, validate the principle.^16^ Given this principle, $p \leqslant \infty p$ always holds, and hence  is false.

## Non-Finean puzzles

The Finean puzzles all involve introduction principles for existential quantification, universal quantification, disjunction or conjunction. Here I wish to introduce new puzzles that still involve introduction principles but of a different nature, and show how they can be handled if we take them to concern weak-first worldly grounding.

One of the four puzzles I introduced in §1 invokes the following general principle, where $\textsf {T}$ is a truth operator:

$$\textsf {T}$$**-Grounding:** $$\phi \prec \textsf {T}\phi $$

I did not previously call this principle into question, but it generates puzzles regardless of its interactions with the introduction principles that are characteristic of the Finean puzzles. Let $\mathfrak {T}$ be a truth-teller sentence, so that $\mathfrak {T} = \textsf {T} \mathfrak {T}$ holds. $\textsf {T}$-Grounding then leads to a violation of $\hbox {Irreflexivity}_\prec$—to *two* violations, indeed: $\mathfrak {T} \prec \mathfrak {T}$ and $\textsf {T}\mathfrak {T} \prec \textsf {T}\mathfrak {T}$.

What is the appropriate reaction if we take ≺ to be weak-first and worldly? Here is a suggestion. We may accept the following principle of “truth introduction” for ⩽:

1 $$\begin{aligned} \phi \leqslant \textsf {T}\phi \end{aligned}$$

Given that ≺ is weak-first, from this principle follows a restricted version of $\textsf {T}$-Grounding, namely:

$$\textsf {T} {\textbf {-Grounding}}^*$$**:** If , then $$\phi \prec \textsf {T}\phi $$

And we may refrain from accepting $\textsf {T}$-Grounding itself. The relevant instance of $\textsf {T}\hbox {-Grounding}^*$ is:

If , then $$\mathfrak {T} \prec \textsf {T}\mathfrak {T}$$

Given that $\mathfrak {T} = \textsf {T} \mathfrak {T}$ and that ≼ is reflexive, the antecedent of this conditional is false and so we cannot detach its consequent. We do not get a violation of $\hbox {Irreflexivity}_\prec$.

Now consider a principle that one may find natural to take on board together with $\textsf {T}$-Grounding:

$$\lnot \textsf {T}$$**-Grounding:** $$\lnot \phi \prec \lnot \textsf {T}\phi $$

This principle and our truth-teller sentence also generate violations of Irreflexivity$_\prec$: $\lnot \mathfrak {T} \prec \lnot \mathfrak {T}$ and $\lnot \textsf {T}\mathfrak {T} \prec \lnot \textsf {T}\mathfrak {T}$. But interestingly, the two principles also give rise to violations of $\hbox {Irreflexivity}_\prec$ independently from the truth-teller sentence. Let $\mathfrak {L}$ be a liar sentence, so that $\mathfrak {L} = \lnot \textsf {T} \mathfrak {L}$ holds. Since $\mathfrak {L} = \lnot \textsf {T} \mathfrak {L}$, $\lnot \mathfrak {L} = \lnot \lnot \textsf {T} \mathfrak {L}$, and so by $\lnot \textsf {T}$-Grounding we get:

2 $$\begin{aligned} \lnot \lnot \textsf {T} \mathfrak {L} \prec \mathfrak {L} \end{aligned}$$

By $\textsf {T}$-Grounding we have:

3 $$\begin{aligned} \mathfrak {L} \prec \textsf {T}\mathfrak {L} \end{aligned}$$

If we follow the mainstream theories of representational grounding and take $p < \lnot \lnot p$ to always hold, we will have $\textsf {T} \mathfrak {L} \prec \lnot \lnot \textsf {T} \mathfrak {L}$. Given (2) and (3) and the transitivity of ≺, we get a violation of $\hbox {Irreflexivity}_\prec$. If we adopt the compelling principle that $p\equiv \lnot \lnot p$ always holds and take ≺ to be worldly, (2) and (3) yield a violation of $\hbox {Asymmetry}_\prec$.

How are we to handle this if we take ≺ to be worldly and weak-first? Given acceptance of (1) above, it is natural to also adopt the dual principle:

4 $$\begin{aligned} \lnot \phi \leqslant \lnot \textsf {T}\phi \end{aligned}$$

From this principle one can derive a restricted version of $\lnot \textsf {T}$-Grounding:

$$\lnot \textsf {T} {\textbf {-Grounding}}^*$$**:** If , then $$\lnot \phi \prec \lnot \textsf {T}\phi $$

And we may refrain from accepting $\lnot \textsf {T}$-Grounding itself. Let us see what we get from this with our truth-teller sentence and our liar sentence.

$\lnot \textsf {T}\hbox {-Grounding}^*$ gets instantiated to:

If , then $$\lnot \mathfrak {T} \prec \lnot \textsf {T}\mathfrak {T}$$

Since $\mathfrak {T} = \textsf {T} \mathfrak {T}$, $\lnot \mathfrak {T} = \lnot \textsf {T} \mathfrak {T}$. Since ≼ is reflexive, the antecedent of this conditional is therefore false, and so we cannot detach its consequent.

Instantiating $\textsf {T}\hbox {-Grounding}^*$ and $\lnot \textsf {T}\hbox {-Grounding}^*$ with the liar sentence, we get:

If , then $$\mathfrak {L} \prec \textsf {T}\mathfrak {L}$$

If , then $$\lnot \mathfrak {L} \prec \lnot \textsf {T}\mathfrak {L}$$

If we could detach the two consequents, then we would get (2) and (3) above and hence a violation of $\hbox {Asymmetry}_\prec$ (assuming $\textsf {T} \mathfrak {L} \equiv \lnot \lnot \textsf {T} \mathfrak {L}$). But none of the antecedents is true if the double negation introduction principle for ⩽, namely principle (1) from §7, holds and if, as is universally taken for granted, ⩽ is transitive. To establish the falsity of the first antecedent, note that given (4), we have $\lnot \mathfrak {L} \leqslant \lnot \textsf {T} \mathfrak {L}$. Since $\mathfrak {L} = \lnot \textsf {T} \mathfrak {L}$, we then have $\lnot \lnot \textsf {T} \mathfrak {L} \leqslant \mathfrak {L}$. By the double negation introduction principle for ⩽ and the transitivity of ⩽, we then get $\textsf {T} \mathfrak {L} \leqslant \mathfrak {L}$, and hence $\textsf {T} \mathfrak {L} \preccurlyeq \mathfrak {L}$. For the second antecedent, note that given (1), we have $\mathfrak {L} \leqslant \textsf {T} \mathfrak {L}$. By the double negation introduction principle for ⩽ and the transitivity of ⩽, we then get $\mathfrak {L} \leqslant \lnot \lnot \textsf {T} \mathfrak {L}$. Since $\mathfrak {L} = \lnot \textsf {T} \mathfrak {L}$, we then have $\lnot \textsf {T}\mathfrak {L} \leqslant \lnot \mathfrak {L}$, and hence $\lnot \textsf {T}\mathfrak {L} \preccurlyeq \lnot \mathfrak {L}$.

So, the proposed account of the grounds of facts of types $\textsf {T}\phi$ and $\lnot \textsf {T}\phi$ escapes the threat posed by truth-tellers and liar sentences.

## No Cantorian threats

Cantor’s theorem states that there is no surjective function from a set to its power set. As a corollary, there is no total injective function from the power set of a set to the set. Focus on sets *of propositions*. Intuitively, there is a function *f* that assigns to each set of propositions *S* *the proposition that every member of* *S* *is true*. (This particular choice of definite description is immaterial; I could have used “the proposition that every proposition belongs to *S*” or “the proposition that *S* is self-identical”, for instance.) Also intuitively, this function is injective: if *S* and *T* are distinct sets of propositions, then it is very tempting to say that *f*(*S*) and *f*(*T*) are distinct since, after all, they are not about the same entities—or, alternatively, they do not have the same constituents. But these intuitions contradict the corollary of Cantor’s theorem: *f*, as it has been characterized, is a total injective function from the set of all sets of propositions to the set of all propositions, and the corollary says that there is no such function. This is the Russell-Myhill paradox (Russell, 1903; Myhill, 1958).

The claim that there is no total injective function from the set of all sets of propositions to the set of all propositions can be formalized in a higher-order language as the claim that no instance of the following schema is true, where *P*, *Q* and *R* are plural sentential variables, ϕ contains only *P* free and ≈ expresses plurality identity:

RM $$\begin{aligned} \forall Q\forall R (\phi [Q/P] = \phi [R/P] \rightarrow Q \approx R) \end{aligned}$$

Here ϕ plays the role of a function *f* taking sets of propositions into propositions; the fact that ϕ[Q/P] is always defined and that the quantifiers are unrestricted captures the claim that *f* is total; and, of course, what (RM) states corresponds to the claim that *f* is injective.

Fritz (2022) argues that widely accepted ground-theoretic principles entail instances of (RM) and hence have to be rejected.^17^ Here I will argue that weak-first worldly grounding escapes this threat from Russell-Myhill.

Fritz provides two derivations of instances of (RM) from ground-theoretic principles, one significantly simpler than the other. The ground-theoretic principles concern the notion of *immediate* partial grounding, a notion we have not met in the previous sections.

The first derivation is based on the following principle, where ⋀ is a generalized conjunction operator and $\prec ^i$ expresses immediate partial grounding:

1 $$\begin{aligned} \forall P \forall p(p \prec ^i \bigwedge P \leftrightarrow p \in P) \end{aligned}$$

In English: the immediate partial grounds of a conjunction are exactly its conjuncts. The principle follows from the natural definition of immediate partial grounding in terms of immediate full grounding and the principle that what immediately grounds a conjunction is the plurality of its conjuncts—a principle which sounds plausible insofar as the notion of immediate full grounding makes sense. The derivation goes as follows. Suppose $\bigwedge Q = \bigwedge R$. Suppose then p∈Q. Then by (1) right-to-left, $p \prec ^i \bigwedge Q$ and so, $p \prec ^i \bigwedge R$. By (1) left-to-right, it follows that p∈R. We show in a similar way that for every p∈R, p∈Q. It follows that Q≈R. Thus, we have established the following conclusion:

$$\begin{aligned} \forall Q \forall R (\bigwedge Q = \bigwedge R \rightarrow Q \approx R) \end{aligned}$$

This is an instance of (RM).

The other derivation, as I stressed, is significantly more complex. Let me just list the principles on which it is based:

2 $$\begin{aligned}&p\prec ^i p \wedge q \qquad q \prec ^i p \wedge q \end{aligned}$$

3 $$\begin{aligned}&r\prec ^i p \wedge q \rightarrow ( r = p \vee r = q) \end{aligned}$$

4 $$\begin{aligned}&p\prec ^i p \vee q \qquad q \prec ^i p \vee q \end{aligned}$$

5 $$\begin{aligned}&r\prec ^i p \vee q \rightarrow ( r = p \vee r = q) \end{aligned}$$

6 $$\begin{aligned}&\forall p (\phi \prec ^i \forall p \phi ) \end{aligned}$$

7 $$\begin{aligned}&\forall q(q \prec ^i \forall p \phi \wedge \forall p (q\ne \phi ) \rightarrow q = t) \end{aligned}$$

8 $$\begin{aligned}&\lnot \forall p\forall q \, p \prec ^i q \end{aligned}$$

(*t* is a propositional constant expressing a suitable “totality fact”.)

In previous sections, I have assumed or suggested at various places that the concepts of grounding we were concerned with are transitive and therefore not concepts of immediate grounding. Be that as it may, it is possible to define immediate concepts in terms of concepts that are not immediate. Here is an easy example. Start with a concept of partial grounding ≺ and define an immediate concept $\prec ^i$ as follows: $p \prec ^i q := p \prec q \wedge \lnot \exists r (p \prec r \wedge r \prec q)$. Alternatively, we may start with a concept of full grounding <, define a concept of immediate full grounding $<^i$ in terms of it in something like the way we just defined $\prec ^i$ in terms of ≺, and then define a concept of immediate partial grounding in the natural way from $<^i$. And there may be other plausible suggestions. What I wish to show in the remainder of this section is that if a concept of immediate partial grounding is worldly—which will be the case for instance if it is defined in the way suggested above in terms of a worldly concept of partial grounding that is not immediate, then Fritz’s derivations of the relevant instances of (RM) are not sound.

Fritz’s derivation based on principles (2)-(8) above can easily be blocked if we suppose that $\prec ^i$ is worldly. Given the compelling view that $(p\wedge p) \equiv p$ always holds, if principle (2) is true and $\prec ^i$ is worldly, then $\prec ^i$ is reflexive. But of course it is not, and therefore (2) must be rejected if $\prec ^i$ is worldly. One can run a similar argument to the conclusion that (4) must be rejected if $\prec ^i$ is worldly, invoking $(p\vee p) \equiv p$ instead of $(p\wedge p) \equiv p$.

(1) with $\prec ^i$ assumed to be worldly can be shown to fail in a similar way if we focus of the degenerate case of conjunctions with only one member: given the compelling view that $\bigwedge p \equiv p$ always holds, $\prec ^i$ can be shown to be reflexive if it is worldly and (1) right-to-left is true. Arguments against (1) involving non-degenerate conjunctions can also be provided. It is plausible to hold that ⋀ is subject to the following principle of “leveling”:^18^

$$\begin{aligned} \forall p\forall q\forall r (\bigwedge \{p, \bigwedge \{q, r\}\} \equiv \bigwedge \{p, q, r\}) \end{aligned}$$

Now consider *p*, *q* and *r* such that *q* is distinct from *r* (so that $\bigwedge \{q, r\}$ is not degenerate) and $\bigwedge \{q, r\}$ is distinct from *p*, *q* and *r*. There surely are cases like this: take “2+2=4” for *p*, “Socrates is human” for *q* and “there are black holes” for *r*, for instance. Now assuming $\prec ^i$ to be worldly, the leveling principle and (1) together preclude such cases.

## Concluding remarks

With the aim of avoiding misunderstandings, let me conclude by mentioning two things I *did not* try to do in this article, adding a few brief comments along the way.

I did not try to establish that weak-first worldly grounding is absolutely puzzle-proof. Doing it would indeed be a tall order. What I did instead is try to show that weak-first worldly grounding escapes the Finean puzzles, Fritz’s puzzle and the other puzzles I have introduced in due course—that is to say, all the logical puzzles of grounding that are known to date. This already has some value, since this is indeed arguably the next best thing.

Nor did I try to argue that, in the light of the discussion of the puzzles, representational grounding ought to be dismissed in favour of worldly grounding. This would perhaps have been expected, since the discussion naturally suggests the following argument, or other similar arguments for the same conclusion that have (a) or something similar as a premise:

- Representational grounding is plagued by paradoxes, worldly grounding is not;
- There is no theoretical role that representational grounding can but worldly grounding cannot play;
- Therefore, we should abandon representational grounding and work only with worldly grounding.

But I am not impressed by such arguments. True, all the puzzles discussed in this paper affect notions of grounding that are representational. But solutions to some of them that do not invoke worldly grounding have been proposed, and it is likely that further solutions of that sort will be put forward. One solution to the Finean puzzles that is of the relevant sort, which I like, consists in simply accepting the cases of self-grounding / of mutual grounding that the corresponding derivations establish—in the spirit of Correia (2014) (see §2 above). Other solutions that preserve the representational character of grounding are discussed in Krämer (2020).

## Footnotes

1 The first puzzles that have been discussed are from Fine (2010a). Other puzzles are discussed in Correia (2011), Krämer (2013) and Donaldson (2017). See Krämer (2020) for an overview and a general discussion.

2 On the factive vs. non-factive distinction, see Fine (2012).

3 Correia (2020) contains a more detailed discussion of the distinction. See also Krämer and Roski 2015, Fine (2017) and Correia and Correia and Skiles (2019). The sort of grounding pluralism I am sympathetic with, which takes there to be both worldly concepts and representational concepts of grounding, all legitimate (see §2), is explicitly endorsed in Correia (2011, 2017b, 2020) and Fine (2017). For a discussion of the possibility of pluralism and of the connections between worldly and representational concepts of grounding under a pluralist view, see Correia (2020): §6 and references therein.

4 Two points in order to avoid misunderstandings. (1) It is important for my purposes that the worldly vs. representational distinction be characterized in terms of closure as I did here rather than in some other, non-equivalent way. (For alternative ways of characterizing the distinction, see, e.g., the three suggestions in Krämer (2021): §4.5.) (2) It is also important for my purposes that worldliness be defined in terms of *factual equivalence* rather than some other notion of equivalence in the vicinity. To illustrate, in Correia (2017a), I study a notion of strict full grounding—let me use $<^*$ for it—that is closed under a notion I dub *propositional equivalence*, which arguably shares lots of logical features with factual equivalence. But $<^*$ is representational rather than worldly: whereas for any truth-evaluable ϕ, ψ and χ, $\phi \wedge (\psi \wedge \chi )$ and $(\phi \wedge \psi ) \wedge \chi$ describe the world as being the same way (or so it appears obvious to me), $\phi , (\psi \wedge \chi ) <^* \phi \wedge (\psi \wedge \chi )$ must hold in the theory explored in Correia (2017a) while $\phi , (\psi \wedge \chi ) <^* (\phi \wedge \psi ) \wedge \chi$ may fail to hold.

5 See Fine (2012) (proof theory) and its companion deRosset and Fine (2023), and Correia (2017a). (Notational remark: Fine (2012) sketches both a proof theory and a semantics for the logic of grounding, which pull in opposite directions: the former fits with a representational conception of grounding and the latter with a worldly conception. I use the bracketed qualifications “proof theory” and “semantics” when referring to Fine (2012) to indicate which part of Fine’s paper I am referring to.)

6 Dorr (2016: 77) is a dissenting voice. It is important in the present context to bear in mind the distinction between disagreeing about the features of factual equivalence on one hand, and attributing different features to distinct relations notions of equivalence on the other hand. Dorr (2016) and I illustrate the first side of the distinction: we disagree about some features of factual equivalence. I (alone!) illustrate the other side of the distinction: I hold that p∨p and *p* are factually equivalent, and likewise for p∧p and *p*, whereas in Correia (2017a), I deny that p∨p and *p* are propositionally equivalent, and likewise for p∧p and *p*, but propositional equivalence is distinct from factual equivalence (see footnote 4). Moreover, my view that neither p∨p nor p∧p is propositionally equivalent to *p* is not in conflict with my view that both p∨p and p∧p are factually equivalent to *p*, because factual equivalence does not entail propositional equivalence (see again footnote 4). A further illustration of the second side of the distinction involves Krämer and me. Krämer (2021) targets a notion of *ground-theoretic equivalence*, which he explicitly defines in terms of an antecedently given notion of grounding. He denies that p∨p and *p* are ground-theoretically equivalent, and likewise for p∧p and *p*, but ground-theoretically equivalence is distinct from factual equivalence (Krämer recognizes this much; see his footnote 34, p. 1658). Moreover, his view that neither p∨p nor p∧p is ground-theoretically equivalent to *p* is not in conflict with my view that both p∨p and p∧p are factually equivalent to *p*, because factual equivalence does not entail ground-theoretic equivalence: to illustrate, whereas $p \wedge (q \wedge r)$ and $(p \wedge q) \wedge r$ are always factually equivalent (or so I claim, see footnote 4), they are not always ground-theoretically equivalent in Krämer’s sense.

7 Fine actually discusses rules for *strict* grounding rather than weak, but his line of argumentation carries over weak grounding.

8 To be accurate, some of the studies just mentioned do not deal with principles involving quantification. I am here assuming, as it is reasonable to do, that the treatment of the quantificational principles described in my characterization of weak-first grounding is the right one given the rest of the characterization.

9 I presented the treatment of the Finean puzzles that I advocate here at the *Truth and Grounds* conference in Ascona (Switzerland) back in 2015.

10 To be accurate, he also introduces some unrestricted introduction principles for ≼, which he then uses to derive restricted principles for a notion of partial strict grounding that is distinct from the one I have been working with all along, whose definition I suggested in §1. Fine (Fine (2012): 53) calls the notion defined in the way suggested in §1*partial strict ground* and the one defined by Lovett *strict partial ground*, and argues that they are distinct—the former being strictly stronger than the latter. I left the latter notion out of the discussion in order to make things a bit simpler, assuming that including it would not make any substantial difference anyway. Likewise, for the sake of simplicity I here pretend that Lovett only works with the ground-theoretic notions at stake in my discussion of the Finean puzzles, but I take it that nothing of substance hinges on this.

11 Fritz works with a factive concept of partial grounding, and accordingly the principle he puts forward is $\forall X \forall p (Xp \rightarrow (Xp \prec EX))$. I stick to my initial decision of working with non-factive concepts of grounding.

12 The principle mixes the Fregean idiom with the standard quantificational idiom, but it could be re-written in fully Fregean fashion and doing so would have no substantial effect on the discussion.

13 Other examples invoke a generalized conjunction operator ⋀, that can take arbitrarily many sentences to make a sentence, and a generalized disjunction operator ⋁ of the same grammatical type. Impure logics of grounding typically do not countenance such operators (but see Correia (2023)), but it is in the spirit of most if not all logics of representational grounding to validate $p \prec \bigwedge p$ and $p \prec \bigvee p$, and it is in the spirit of most if not all logics of worldly grounding to validate $p \equiv \bigwedge p$ and $p \equiv \bigvee p$.

14 Batchelor (2010), Schnieder (2011), Fine (2012) (proof theory), Correia (2014, 2015, 2017a, 2021), Poggiolesi (2016), Krämer (2018), Korbmacher (2018a, 2018b), deRosset and Fine (2023).

15 The view is validated in Fine (2012) (semantics), Correia (2010, 2016). See Ramsey (1927) for a compelling argument for the view, and Dorr (2016) for sympathetic comments.

16 The references are in §4. Assuming that < is both weak-first and worldly, one may argue for the principle as follows. Given that < is weak-first and worldly, ⩽ must also be worldly: given condition (C), it is hard to see how < could be worldly without ⩽ also being worldly. Now given that ⩽ is reflexive and that $p \equiv \lnot \lnot p$ always holds (see above), $p \leqslant \lnot \lnot p$ always holds.

17 To be accurate, Fritz invokes two schemas rather than one, whose quantifiers are restricted to truths. He needs to do it that way because he works with a factive notion of grounding. Here I follow my general policy of working with non-factive notions, which allows me to invoke just one schema, (RM), instead of Fritz’s two schemas. This policy will allow me to work with principles that are sensibly simpler than Fritz’s in the discussion below. I will not bother to state the differences and will speak as if Fritz invoked the simpler principles.

18 The term is from Fine (2010b). Note that the principle is formulated in terms of factual equivalence rather than identity. The principle follow from the associativity principle of the system for factual equivalence defined in Correia (2023): 589.
